# Precursor-Derived Mo_2_C/SiC Composites with a Two-Dimensional Sheet Structure for Electromagnetic Wave Absorption

**DOI:** 10.3390/ma18071573

**Published:** 2025-03-31

**Authors:** Yang Li, Wen Yang, Jipeng Zhang, Yongzhao Hou, Guangwu Wen, Guodong Xin, Meixian Jiang, Yongqiang Ma

**Affiliations:** 1 School of Materials Science and Engineering, Shandong University of Technology, Zibo 255000, China; 15650323857@163.com (Y.L.); wengw@sdut.edu.cn (G.W.); 2 School of Transportation and Verhicle Engineering, Shandong University of Technology, Zibo 255000, China; zhangjp@sdut.edu.cn; 3 Weifang Kaihua Silicon Carbide Micropowder Co., Ltd., Weifang 261207, China; 867047607@163.com; 4 Yantai Glass Coating Micro-Nano Imprinting Technology Innovation Center, Conor Glass Science & Technology Co., Ltd., Yantai 265700, China; jiangmeixian@conorgt.com (M.J.); mayongqiang@conorgt.com (Y.M.)

**Keywords:** Mo_2_C/SiC, polymer-derived ceramics (PDC), two-dimensional sheet structure, wave absorption

## Abstract

Precursor-derived silicon carbide (SiC) ceramics have been widely used as absorbing materials, but the residual carbon sink produced by ceramicization limits their application under high-temperature and oxygen-containing conditions, such as the nozzle or jet vane of high-speed aircraft. In this paper, a novel molybdenum carbide/silicon carbide (Mo_2_C/SiC) microwave-absorbing ceramic with a two-dimensional sheet structure was obtained through the pyrolysis of polycarbosilane-coated molybdenum sulfide (PCS@MoS_2_). The results indicate that addition of an appropriate amount of MoS_2_ can react with the free carbon generated during the pyrolysis of PCS, thereby reducing the material’s carbon content and forming Mo_2_C. Concurrently, the layered structural characteristics of MoS_2_ are utilized to create a two-dimensional composite structure within the material, which enhances the material’s absorption vastly. The as-prepared Mo_2_C/SiC ceramics sintered at 1300 °C exhibit a minimum reflection loss (RL_min_) of −46.49 dB at 8.96 GHz with a thickness of 2.6 mm. Additionally, the effective absorption bandwidth (EAB) of Mo_2_C/SiC spans the entire X-band (8–12 GHz) due to the combined effect of multiple loss mechanisms.

## 1. Introduction

In recent years, as the requirements for wave-absorbing materials have become increasingly stringent, the preparation of ceramic-based wave-absorbing materials via precursor-derived ceramic (PDC) has gained much more attention [1,2,3,4,5]. The PDC method can be utilized to achieve the adjustability of the composition, structure, and properties of the product by designing the precursor, thereby meeting the requirements for different material properties. Various wave-absorbing ceramics have been prepared by the PDC route, such as SiCO [6,7], SiC [8,9], SiCN [10,11,12], and SiBCN [13,14,15].

However, the main chain or branch chain of the ceramic precursor molecule contains many carbon atoms, resulting in the conversion of the precursor to the ceramic being mostly carbon rich. The presence of carbon can also effectively improve the wave-absorbing properties of the material [16,17,18,19]. For example, Mo et al. prepared carbon rich porous SiCO ceramics by combining the hydrothermal method and the PDC method [20]. The findings revealed that the SiCO produced through annealing at 1400 °C possesses multiple crystalline phases and a substantial quantity of free carbon. Moreover, it develops an interconnected conductive network and a wealth of heterogeneous phase interfaces, thereby significantly boosting the material’s wave-absorbing capabilities. Nevertheless, such carbon-rich materials cannot operate efficiently for prolonged durations in oxygen-rich settings owing to the instability of carbon under high-temperature conditions [21].

Therefore, to prepare a high temperature absorbing material, it is necessary to convert the carbon in the precursor into a high temperature stable phase. Feng [22] used ferric acetylacetonate (FA) to modify the precursor, and PDCs-SiCN (Fe) ceramics containing the Fe_3_C phase were prepared by sintering at 1100 °C. PDCs-SiCN (Fe) ceramics with 50 wt% of FA addition have a wide effective absorption bandwidth (EAB) in 5.1–8.4 GHz. Wei [23] achieved SiCTi wave-absorbing ceramics with TiC, exhibiting outstanding performance, through the addition of tetrabutyl titanate as a modifier to the precursor. The results showed that the 3% tetrabutyl-titanate-modified PCS sintered SiC effectively, reducing the C content of the material. The minimum reflection loss (RL_min_) of SiCTi is −16 dB at 8.9 GHz, and the EAB is 4.2 GHz (in the whole X-band) when the thickness is varied from 1.7 to 2.4 mm. In particular, incorporating the molybdenum (Mo) element into the precursor allows for the modification of the material. This confirms that Mo can interact with silicon (Si) and carbon (C) to form diverse phases, such as molybdenum carbide, molybdenum silicide, and silicon–carbon–molybdenum alloys. These phases expand the options for choosing wave-absorbing components. [24,25].

How to introduce molybdenum in the precursor with a molybdenum source using molybdenum powder or molybdenum oxide or molybdenum sulfide is a key problem. In our early research, a SiCO@BN 2D wave-absorbing material with better impedance matching and resonant cavity structure was prepared [26]. Similarly, the two-dimensional PCS@graphene [27], PCS@BN [28] were prepared for wave absorption. The newly generated wave-absorbing materials have a unique two-dimensional structure already rich in phase interfaces, which greatly improves the wave-absorbing properties of the materials.

Hence, in this paper, molybdenum disulfide (MoS_2_) is combined with the precursor to prepare a wave-absorbing material with a two-dimensional structure. In the high-temperature sintering process, MoS_2_ reacts with the PCS to generate molybdenum carbide (mainly Mo_2_C). Because of two-dimensional MoS_2_, these Mo_2_C/SiC materials contain a greater quantity of phase interfaces, which enhances the interfacial polarization and then improves the wave-absorbing property.

## 2. Experimental

### 2.1. Materials

Polycarbosilane (PCS) was provided by the Institute of Chemistry, Chinese Academy of Sciences (Beijing, China). Molybdenum disulfide (MoS_2_, 99.5%) and tetrahydrofuran (C_4_H_8_O, 99.5%) were purchased from the McLean Reagent platform.

### 2.2. Preparation of Mo_2_C/SiC Ceramics

**Figure 1:**
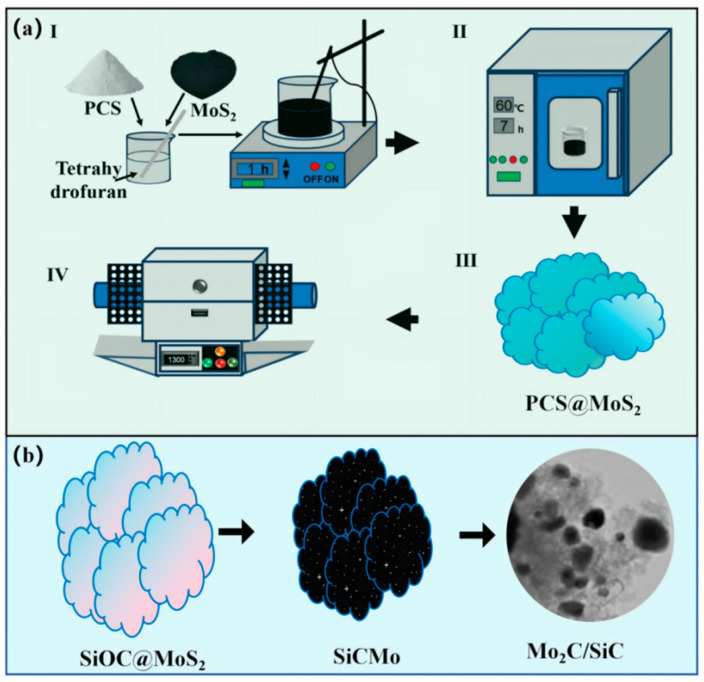
(**a**) The preparation diagram, (**b**) Composition change process of Mo_2_C/SiC ceramic.

This experiment adopts the PDC method to prepare Mo_2_C/SiC ceramic material. The specific process is shown in Figure 1. Firstly, the required solid PCS and MoS_2_ were added into tetrahydrofuran. After ultrasonic dispersion and uniform stirring for 0.5 h, the PCS was completely dissolved. In this process, the MoS_2_ nanosheets are uniformly dispersed in the solution, and the nanosheets gradually become thinner. Due to the encapsulation of PCS, the nanosheets will no longer agglomerate due to van der Waals’ force. The tetrahydrofuran was removed by vacuum distillation to obtain fluffy PCS@MoS_2_ powder. The four PCS@MoS_2_ were prepared with the mass ratio of PCS:MoS_2_ at 1:1, 2:1, 3:1, and 4:1, respectively. Finally, the prepared PCS@MoS_2_ were pyrolyzed at 1300 °C for 3 h under nitrogen (N_2_), and the samples of different ratios were named P1, P2, P3, and P4, respectively.

### 2.3. Characterization

X-ray diffraction spectroscopy (XRD, Rigaku XRD 2500, Tokyo, Japan) is mainly used to study the composition of materials, with Cu Kα radiation (30.0 kV, 20.0 mA) in the 2θ range from 10–90°. Infrared spectroscopy (FI-IR, Thermo Nicolet AVATAR 370, Richmond Scientific, Chorley, UK) was used to analyze the precursor properties before sintering, within the spectral range spanning from 4000 to 450 cm^−1^. Thermogravimetric analysis (TG, NETZSCH STA 449 F3, Selb, Germany) was used to study the material quality changes during the sintering process(RT−1000 °C) and then analyze the reaction at each stage at a heating rate of 10 °C/min in a N_2_ atmosphere. Raman spectroscopy (Alpha300R, Laser wavelength 532 nm, Oxford Instruments, Abingdon, UK) was used to analyze the degree of graphitization of materials within the spectral range spanning from 2500 to 500 cm^−1^. X-ray photoelectron spectroscopy (XPS, Thermo Fisher 0ESCALAB 250Xi, Waltham, MA, USA) was used to further analyze the chemical composition of the material. The surface morphology of the materials was observed by scanning electron microscopy (SEM, Zeiss Gemini 300, Jena, Germany). Transmission electron microscopy (TEM, JEM-F200, JEOL, Tokyo, Japan) was used to observe the microstructure of the materials.

The vector network analyzer (Agilent Technologies N5280A, Santa Clara, CA, USA) was used to measure the electromagnetic parameters of the absorber ring via the standard coaxial-line technique in the test range of 8 to 12 GHz (X-band). With paraffin wax serving as the foundational support at 55 wt%, an absorber ring(Φ_in_ = 3.04 mm, Φ_out_ = 7 mm) is produced with a thickness of 2 to 4 mm.

## 3. Result and Discussion

### 3.1. Characterizations of Mo_2_C/SiC

**Figure 2:**
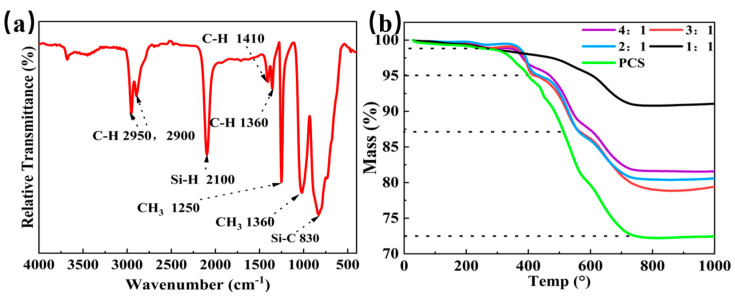
(**a**) IR spectrum and (**b**) TG curves of PCS@MoS_2_.

As shown in Figure 2a, the PCS@MoS_2_ exhibits obvious characteristic peaks at 690~860 cm^−1^, 1020 cm^−1^, 1250 cm^−1^, 2900, and 2950 cm^−1^, which are consistent with the Si-C bond, Si-C-Si bond, Si-H bond, and C-H bond of the precursor (PCS) [29,30]. This indicates that PCS does not react with MoS_2_ in unpyrolyzed PCS@MoS_2_. After pyrolysis at a high temperature, PCS will release the organic phase and gradually transform into SiC, while PCS@MoS_2_ shows different rules on the TG curve in Figure 2b. Compared to PCS(mass loss at 72.5%), the mass loss of the four PCS@MoS_2_ decreased apparently, while the ceramic yield of P1~P4 is maintained at 91.09%, 80.62%, 79.45%, and 81.56%, respectively. This shows that the doping of MoS_2_ will affect the ceramic transformation of PCS. The pyrolysis weight loss of PCS@MoS_2_ is divided into three stages [31,32]. During the 200–400 °C stage, some small molecules in PCS do not participate in cross-linking and curing and will be removed by gas. From the DTG curve(Supporting Information Figure S1), the quality loss of the attachment is the fastest at 380–390 °C DTG curves, which attributed to the low molecular weight oligomers. In the range of 400–600 °C, the PCS molecular chain breaks down to form smaller molecular chain fragments, especially in the 520–500 °C in the DTG curve. At 600–800 °C, the branched chain in PCS will break and generate methane (CH_4_), which is derived from the cleavage of-Si-CH_3_ on the branched chain of PCS. At higher temperatures, PCS is completely converted into amorphous SiC, and it will react with molybdenum sulfide to form a Mo_2_C/SiC alloy phase.

**Figure 3:**
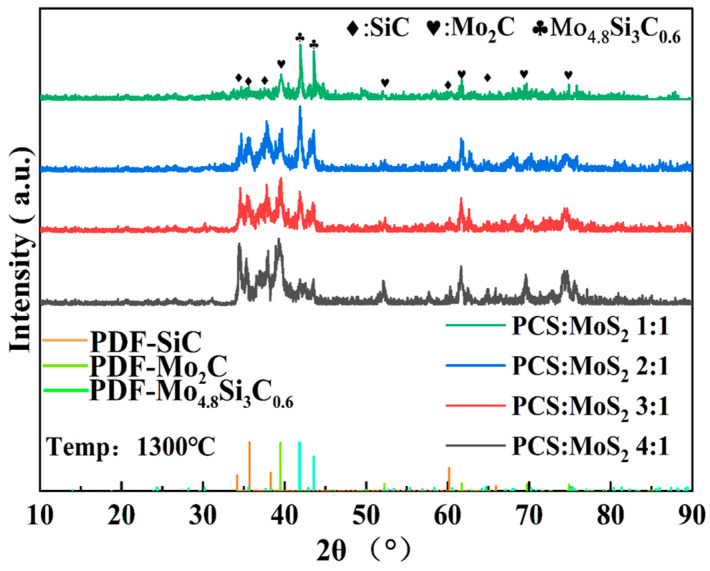
XRD pattern of Mo_2_C/SiC.

The XRD in Figure 3 shows the crystalline phase of the Mo_2_C/SiC derived from PCS@MoS_2_ after sintering at 1300 °C. Firstly, the sintered samples exhibit distinct diffraction peaks at angles of 35.6°, 60.2°, and 65.1°, which correspond to the (111), (110), and (012) crystal planes of β-SiC [33]. The peaks are at 39.4°, 52.1°, 61.5°, 69.6°, and 74.6°, which are associated with the (101), (102), (110), (103), and (112) crystal planes of β-Mo_2_C [34]. Furthermore, two diffraction peaks are observed at 41.8° and 43.6°, which correspond to the characteristic peaks of the Mo-Si-C alloy (Mo_4_._8_Si_3_C_0_._6_) [25]. With the increase in PCS ratio, the diffraction peaks corresponding to SiC and Mo_2_C are enhanced, while the peak intensity of Mo_4_._8_Si_3_C_0_._6_ is weakened. This indicates that the intermediate Mo-Si-C alloy phase exists during the pyrolysis process. With the increase in the ratio of carbon and silicon in the pyrolysis products, the Mo-Si-C alloy phase is induced to yield SiC and Mo_2_C phases.

In detail, this two-dimensional flaky PCS@MoS_2_ forms amorphous SiOC ceramic-coated MoS_2_ (SiOC@MoS_2_) in the low-temperature pyrolysis stage. As the temperature further increases, SiOC and MoS_2_ gradually react to form a Si-C-Mo alloy phase, which could continue to form SiC and Mo_2_C. With the increase in PCS, the MoS_2_ in the pyrolysis products will gradually decrease, and the SiC and Mo_2_C phases will gradually increase. The change in ceramic phase composition is shown in Equation (1).

(1) $$PCS@MoS_{2}\to SiOC@MoS_{2}\to Mo-Si-Calloy\to SiC+{Mo}_{2}C$$

**Figure 4:**
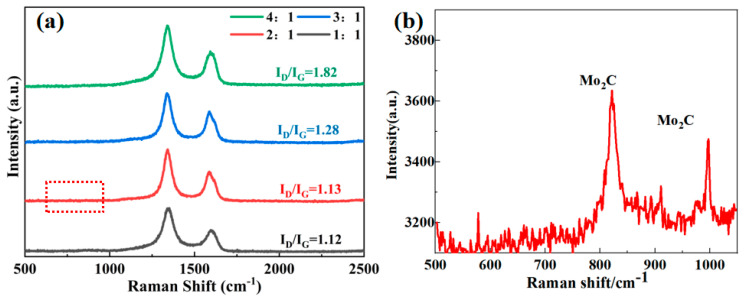
Raman spectrum of Mo_2_C/SiC. (**a**) Raman spectrum of Mo_2_C/SiC, (**b**) Amplification of Raman spectra in the red dotted box in Figure 4a (500–1000 cm^−1^).

The Raman spectra of PCS:MoS_2_ from 1:1 to 4:1 are shown in Figure 4. Typically, the vibrations associated with sp2-hybridized carbon and the presence of internal defects and disorder in the crystal structure are shown in the D-band (1350 cm^−1^) and G-band (1580 cm^−1^) of the Raman spectra [25,35]. The I_D_/I_G_ values of P1, P2, P3, and P4 are 1.82, 1.28, 1.13, and 1.12, respectively, indicating that the amount of disordered carbon rises in tandem with the increase in the precursor PCS. A higher I_D_/I_G_ ratio signifies a greater abundance of carbon defects within the Mo_2_C/SiC. This phenomenon occurs because the pyrolysis products contain an excess of carbon, and only a small amount of carbon and Mo react to form Mo_2_C. Moreover, when the region from 500–1000 cm^−1^ in the Raman spectrum is magnified, a distinct peak at 821 cm^−1^ and 992 cm^−1^ for Mo_2_C will become apparent in Figure 4b.

### 3.2. Microstructure of Mo_2_C/SiC

**Figure 5:**
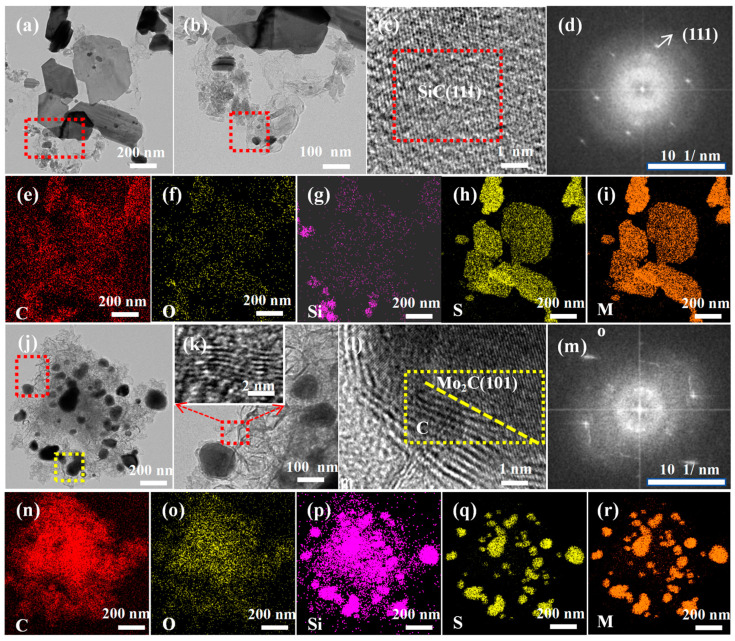
(**a**,**b**) Morphology of P1, (**c**) lattice fringe of P1, (**d**) diffraction pattern of p1, (**e**–**i**) element surface scanning of P1, (**j**) morphology of P3, (**k**,**l**) lattice fringe of P3, (**m**) diffraction pattern of P3, (**n**–**r**) element surface scanning of P3.

The microstructure and composition of Mo_2_C/SiC ceramics were investigated using transmission electron microscopy (TEM) in Figure 5. It is evident that both P1 and P3 samples retain a two-dimensional sheet structure, with the size of the P1 and P3 nanosheets ranging from 400–800 nm (Figure 5a,b,j). It is apparent that the P3 nanosheets are generally thinner, a result of the increased addition of PCS during the preparation of P3. In the formation of PCS@MoS_2_, PCS molecules impede the stacking of molybdenum sulfide sheets, which facilitates the dispersion of the molybdenum sulfide sheets. Elemental surface scans reveal that both nanosheets contain several elements: C, O, Si, S, and Mo (Figure 5e–i,n–r), suggesting that the prepared P1 and P3 maintain the composition of SiOC@ MoS_2_ elements. The surface scan image areas of SiOC cover sulfur and molybdenum elements, indicating that the molybdenum sulfide nanosheets are encapsulated by SiOC. Although the XRD analysis results suggest that SiOC and molybdenum sulfide form an alloy phase and further precipitate silicon carbide and molybdenum carbide, this reaction is incomplete. The molybdenum sulfide phase is present in both P1 and P3. The wrinkles of the MoS_2_ layered structure are visible in Figure 5j, and the lattice fringes of MoS_2_ are seen in Figure 5k, with a crystal plane spacing of 0.62 nm. However, the molybdenum sulfide in P1 is significantly larger than that in P3, indicating that molybdenum sulfide in P3 (with more PCS) is more extensively involved in the reaction, forming more silicon carbide and molybdenum carbide. Figure 5c,d display clear lattice fringes and diffraction patterns of the SiC (111) crystal plane, with a crystal plane spacing of 0.25 nm. The (101) crystal plane of Mo_2_C and the lattice fringes and diffraction patterns of unreacted surplus carbon are visible in Figure 5l,m, with interplanar spacings of 0.23 nm and 0.34 nm, respectively.

**Figure 6:**
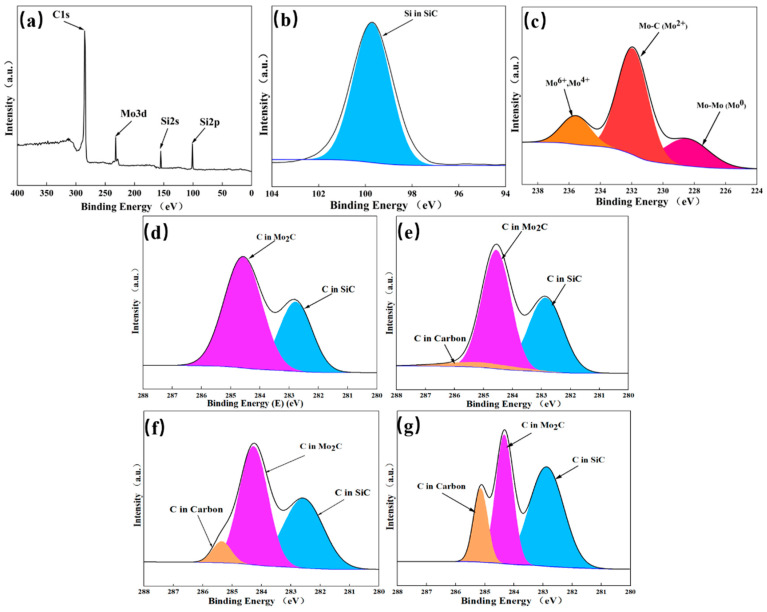
Sample XPS diagram: (**a**) total energy spectrum, (**b**) Si2p, (**c**) Mo3d, (**d**) C1s of the P1 sample, (**e**) C1s of the P2 sample, (**f**) C1s of the P3 sample, (**g**) C1s of the P4 sample.

To further analyze the composition of Mo_2_C/SiC, XPS tests were performed on P1, P2, P3, and P4, and Figure 6a displays four characteristic peaks corresponding to Si2p, Si2s, C1s, and Mo3d. The Si2p spectrum in Figure 6b shows a single peak at 99.5 e V [23], indicating that only the SiC phase exists. The Si-C-Mo alloy phase in the pyrolysis product is a solid solution and does not form a chemical bond. The peaks of Mo3d spectra at 228.7 and 232 eV were typically attributed to Mo^3+^ in Mo_2_C in Figure 6c [36,37], while the peaks at 229.7 and 233.0 eV were attributed to Mo^4+^ and the peaks at 232.8 and 236 eV were Mo^6+^, which were assigned to the surface oxidation of Mo_2_C and unreacted MoS_2_. Figure 6d–g depict the C1S spectra of P1–P4, and three typical C-Si [9,23], C-Mo [36], and C-C [23] binding peaks are located near 284.5 eV, 283 eV, and 285 eV, respectively. The analysis of the P1 sample shows that there is no obvious C-C [23] binding peak, indicating that the Mo doping amount is relatively high at this ratio, and the pyrolytic carbon is fully combined with Mo to form Mo_2_C. With the increase in PCS ratio, the C-C peak increases relatively little from P1 to P4.

### 3.3. Electromagnetic Properties of the Mo_2_C/SiC

To investigate the electromagnetic wave absorption properties of Mo_2_C/SiC composites, the reflection loss (RL) was calculated based on the transmission line theory and the Equations (2) and (3) [38,39].

(2) $$RL\left(dB\right)=20lg|\frac{Z-1}{Z+1}|$$

(3) $$Z=\frac{Z_{in}}{Z_{0}}=\sqrt{\frac{\mu_{r}}{\varepsilon_{r}}}tanh(j\frac{2\pi fd}{c}\sqrt{{\mu_{r}\cdot \varepsilon }_{r}})$$

Here, *Z_in_*, *Z*_0_, *μ_r_*, and *ε_r_* denote the input impedance, intrinsic impedance, complex permittivity, and complex permeability of the absorber, respectively. The variable *f* represents the frequency of the electromagnetic wave, *d* signifies the thickness of the absorbing coating layer, and *c* is the speed of light in a vacuum.

**Figure 7:**
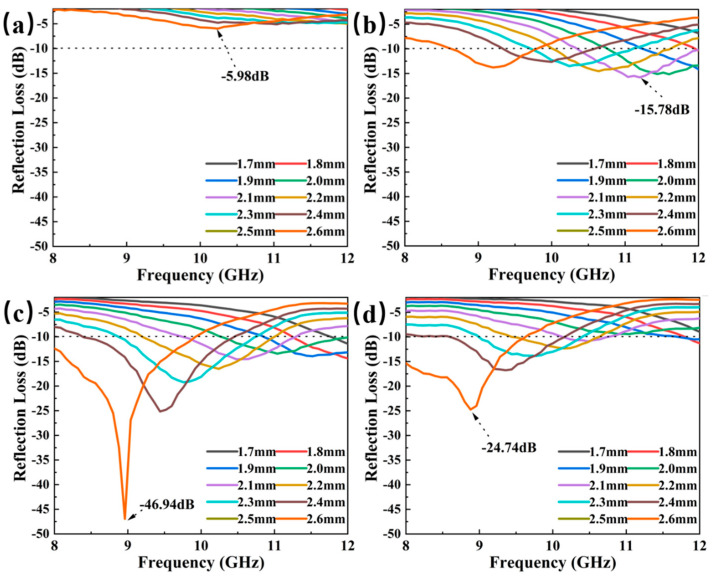
Reflection loss of P1 (**a**), P2 (**b**), P3 (**c**), and P4 (**d**).

In Figure 7, it can be seen that the electromagnetic wave absorption properties of Mo_2_C/SiC composites are very susceptible to the microstructure and composition changes of the materials. As shown in Figure 7a, when the thickness is 2.6 mm, the minimum RL (RL_min_) of P1 is −5.96 dB, and the overall performance is weak. This is because P1 contains more MoS_2_, less nano-SiC and Mo_2_C formed in situ, and the interfacial polarization is weak. As depicted in Figure 7b,c, the imaginary component of the dielectric loss and the tangent of the dielectric loss represent the lowest values across the four sets of samples. However, with the increase in PCS content in the preparation of Mo_2_C/SiC, the absorbing properties are gradually enhanced. Comparatively, the composite electromagnetic wave absorption performance of P2, P3, and P4 has been significantly improved. For P2 (Figure 7b), the RLmin reaches −15.78 dB with a matching thickness of 2.1 mm, and the effective absorption bandwidth (EAB) spans 2.23 GHz (from 9.77 to 12 GHz). Notably, for P3 (Figure 7c), the RL_min_ is −46.94 dB at a thickness of 2.4 mm, while the EAB covers 2.51 GHz (from 8.48 to 11 GHz). Furthermore, with a thickness ranging from 1.7 to 2.6 mm, full-band absorption from 8 to 12 GHz can be achieved due to the cumulative layer thickness effect [23]. For P4 (Figure 7d), the RL_min_ and the EAB is −24.74 dB and 2 GHz at a thickness of 2.6 mm, respectively. Therefore, among the Mo_2_C/SiC composite materials, P3 exhibits the best electromagnetic wave absorption performance. To better compare the absorption properties, various Mo_2_C-based absorbing materials were analyzed in Table 1. It was discovered that these materials, primarily composed of Mo_2_C and C, are prone to oxidation at high temperatures, significantly limiting their application in aerobic high-temperature environment [25,34,40,41,42,43,44]. The Mo_2_C/SiC synthesized in this study not only exhibits high-temperature and oxidation resistance but also demonstrates outstanding wave absorption capabilities.

Additionally, the peak (RL_min_) of the Mo_2_C/SiC waves shifts towards lower frequencies with increasing thickness, as explained by the quarter-wavelength attenuation theory depicted in Figure 7, which is described by Equation (4) [23]:

(4) $$t_{m}=\frac{n\lambda }{4}=\frac{nc}{4f_{m\sqrt{|\mu_{r}||\varepsilon_{r}|}}}\;n=1,3,5$$

where *t_m_* represents the thickness of the absorbers, *λ* denotes the wavelength, *c* is considered the speed of light in a vacuum, *f_m_* signifies the frequency at the peak, |*μ_r_*| and |*ε_r_*| stand for the moduli of the *ε_r_* and *μ_r_* at *f*_m_, respectively, and *n* is an odd number (1, 3, 5…).

**Figure 8:**
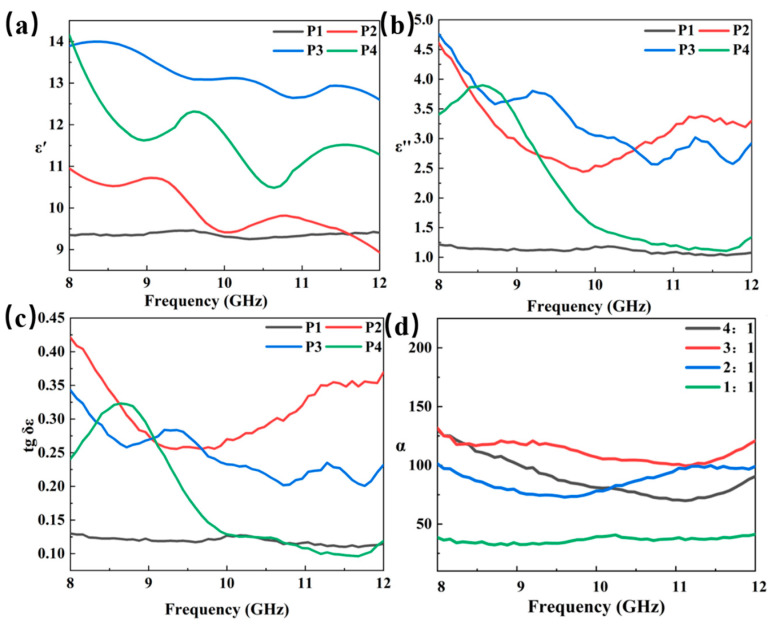
Comparison of permittivity: (**a**) ε’, (**b**) ε″, (**c**) tanδε, and (**d**) Attenuation factor (α).

The electromagnetic wave absorption performance of absorbers primarily depends on their electromagnetic parameters, including the complex permittivity (ε_r_ = ε′ − jε″) [45] and permeability (μ_r_ = μ′ − jμ″) [46]. To observe the variations in the electromagnetic wave absorption performance of Mo_2_C/SiC ceramics, the electromagnetic parameters of four samples were compared in Figure 8. Since Mo_2_C/SiC ceramics lack magnetic properties, their electromagnetic wave absorption performance primarily relies on their complex permittivity.

Within the 8–12 GHz frequency range, the real permittivity (ε′) of P1 exhibits minimal fluctuation, remaining essentially stable between 9.3–9.5. In contrast, the ε′ of P2, P3, and P4 experiences a slight increase, with P3 showing the most significant rise, ranging from 13.8–14. Regarding the imaginary permittivity (ε″), P1 maintains a value below 1.5 overall. However, P2 and P3 have imaginary parts exceeding 2.5 within the 8–12 GHz range, indicating an enhanced loss capacity. P4 only displays a higher imaginary part in the low-frequency region. This pattern is similarly reflected in the dielectric loss behavior. This occurrence is due to the rise in PCS, which leads to the in situ formation of various nanocrystalline phases, such as SiC and Mo_2_C. These abundant phase interfaces and defects significantly enhance the polarization effect, leading to a substantial improvement in the imaginary part.

**Figure 9:**
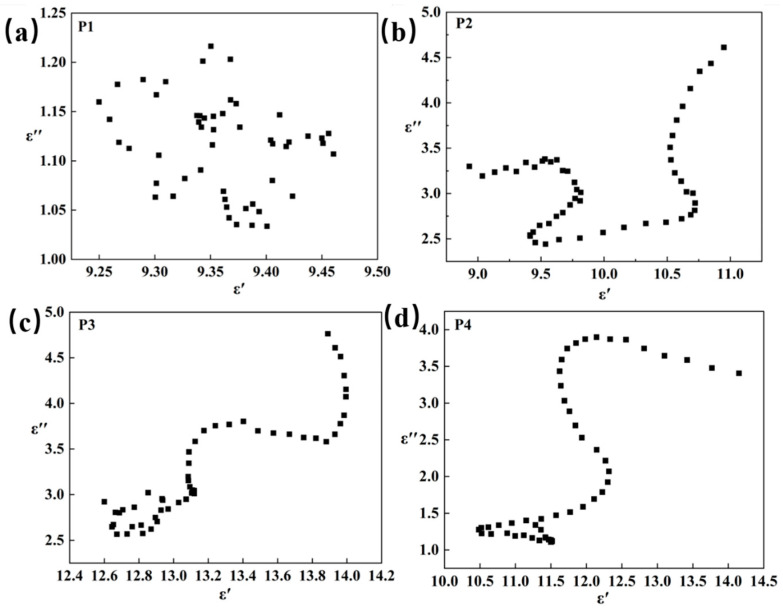
Cole-Cole diagram of P1 (**a**), P2 (**b**), P3 (**c**), and P4 (**d**).

Within most frequency ranges, the ε′ and ε″ values for samples P1 to P4 initially increase and then decrease. This phenomenon indicates that polarization loss significantly impacts the dissipation of electromagnetic energy. The polarization loss of Mo_2_C/SiC ceramics is primarily determined by Debye relaxation polarization [47], as depicted by Equations (5)–(7) [17,45].

(5) $$\varepsilon_{r}=\varepsilon^{\prime }-{j\varepsilon }^{\prime \prime }=\varepsilon_{\infty }+\frac{\varepsilon_{s}-\varepsilon_{\infty }}{1+j2\pi f\tau }$$

(6) $$\varepsilon^{\prime }=\varepsilon_{\infty }+\frac{\varepsilon_{s}-\varepsilon_{\infty }}{1+{\left(2\pi f\right)}^{2}\tau^{2}}$$

(7) $$\varepsilon^{\prime \prime }=\frac{2\pi f\tau (\varepsilon_{s}-\varepsilon_{\infty })}{1+{\left(2\pi f\right)}^{2}\tau^{2}}$$

where *ε*_∞_ represents the relative dielectric permittivity at infinite frequency, *ε_s_* denotes the static dielectric permittivity, *f* is the frequency, and *τ* signifies the polarization relaxation time. Consequently, *ε*′ and *ε*″ are defined by Equations (6) and (7), respectively. According to the aforementioned formula, as the frequency increases, the dielectric constant should exhibit a gradual decrease. However, the peak at a specific frequency may be associated with the intrinsic resonance frequency. Furthermore, if Mo_2_C/SiC adheres to the Debye relaxation polarization equation, the real and imaginary components of the dielectric constant curve will exhibit a semicircular shape. As observed in Figure 9, Mo_2_C/SiC displays multiple semicircles, suggesting the presence of multiple relaxation polarization processes.

In addition, there is another parameter that can be used to judge the absorbing ability from another perspective, such as the attenuation constant (α), which can be calculated by Equation (8) [48].

(8) $$\alpha =\frac{\sqrt{2\pi f}}{c}{\left[\left(\mu^{\prime \prime }\varepsilon^{\prime \prime }-\mu^{\prime }\varepsilon^{\prime }\right)+\sqrt{{\left(\mu^{\prime \prime }\varepsilon^{\prime \prime }-\mu^{\prime }\varepsilon^{\prime }\right)}^{2}+{\left(\mu^{\prime }\varepsilon^{\prime \prime }+\mu^{\prime \prime }\varepsilon^{\prime }\right)}^{2}}\right]}^{\frac{1}{2}}$$

As shown in Figure 6d, sample P3 demonstrates the highest α among the four types of Mo_2_C/SiC ceramics with the value above 120, indicating its superior electromagnetic wave attenuation capability.

**Figure 10:**
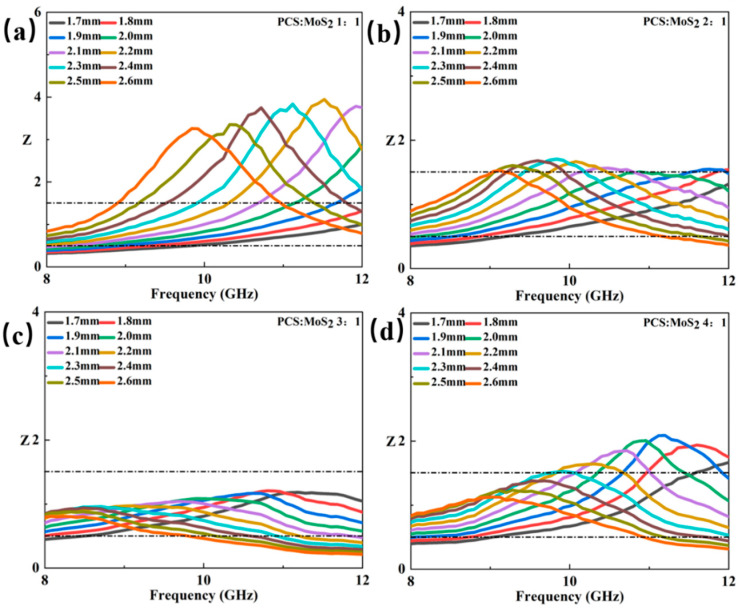
Impedance matching diagram (Z) of P1 (**a**), P2 (**b**), P3 (**c**), and P4 (**d**).

An ideal absorbing material should have good impedance matching in addition to strong loss ability, which can ensure that the electromagnetic wave is not reflected off. Impedance matching is typically indicated by the value of impedance matching (Z) [49]. The closer this value is to 1, the better the impedance matching condition of the absorber. As shown in Figure 10, in the four samples of Mo_2_C/SiC ceramics, the P1 and P4 values deviate from 1 by a large margin. The Z values of P2 and P3 are closer to 1, indicating that the sintered samples with this ratio have excellent impedance matching, which is due to its unique card-like stacking form composed of two-dimensional nanosheets.

### 3.4. Absorbing Mechanism

**Figure 11:**
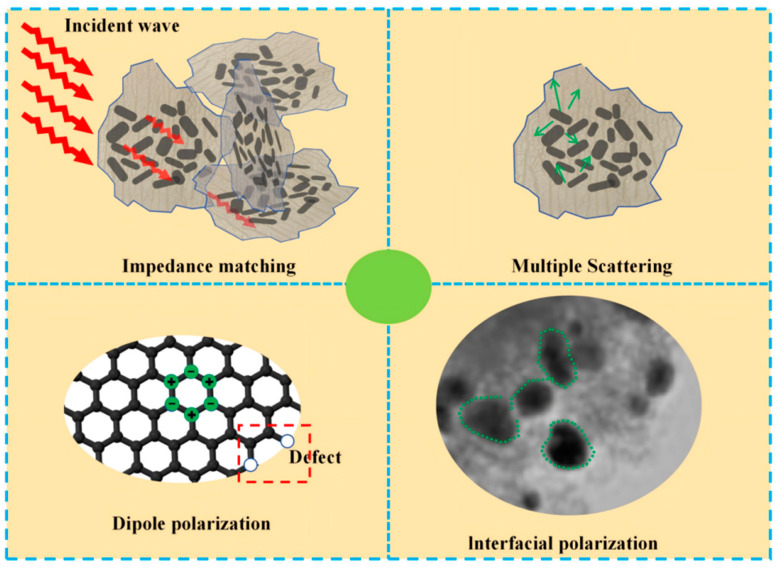
Absorbing mechanism of Mo_2_C/SiC.

The absorption mechanism of Mo_2_C/SiC primarily involves good impedance matching, multiple scattering, dipole polarization, and interface polarization, as depicted in Figure 11. The Mo_2_C/SiC material features a distinctive two-dimensional sheet structure that forms a card-like stacking arrangement, with the stacking gaps contributing to the material’s excellent impedance matching. When electromagnetic waves pass through Mo_2_C/SiC, many more waves penetrate the material rather than being reflected. From a TEM perspective, numerous newly formed nano-SiC and Mo_2_C particles are observed on the two-dimensional structure of Mo_2_C/SiC, which enhance the multiple scattering of electromagnetic waves and aid in the gradual dissipation of these waves within the card-like structure. Notably, Mo_2_C/SiC produces a significant amount of nanocrystalline phases, which greatly benefit the aggregation of charge and enhance the material’s dielectric loss capabilities. Furthermore, the presence of numerous defects and dangling bonds in the disordered carbon exacerbates the uneven distribution of positive and negative charges, thereby intensifying the dipole polarization.

## 4. Conclusions

In this paper, a Mo_2_C/SiC ceramic wave-absorbing material is prepared by using PCS as a precursor and MoS_2_ as a modified material. After high-temperature ceramicization, PCS and MoS_2_ react in situ to form Mo_2_C/SiC alloy, and then the stable phases of Mo_2_C and SiC are precipitated, which solves the surplus carbon in the precursor conversion process. This new Mo_2_C/SiC features a graphite-like lamellar two-dimensional composite structure that enhances the material’s interfacial polarization loss capability and improves its electromagnetic wave loss capability. By adjusting the ratio of raw materials, sintering temperature, and filler ratio, the optimal conditions of Mo_2_C/SiC ceramics were obtained. The peak value of −46.49 dB appeared at 8.96 GHz and 2.6 mm, and the EAB could cover the whole X-band (8–12 GHz). Overall, the Mo_2_C/SiC wave-absorbing ceramics feature a unique lamellar two-dimensional composite structure that offers excellent electromagnetic wave absorption. In addition, the prepared ceramic absorbing materials possess excellent high-temperature and oxidation resistance, enabling their use as stealth coatings in extreme environments, such as the tail nozzles and jet vanes of high-speed aircraft.

## Figures and Tables

**Table 1:** The RL_min_ and EBW of the samples prepared in this experiment were compared with other types of Mo_2_C absorbing materials.

Absorbers	RL_min_/Thickness	EBW/Thickness	Refs.
Mo_2_C/SiC	−46.94 dB/2.6 mm	4 GHz/2.6 mm	This work
Mo_2_C/FeCo/NC	−56.03 dB/3.4 mm	10.27 GHz/3.4 mm	[40]
FCN-Mo_2_C	−36.80 dB/2.9 mm	7.04 GHz/2.9 mm	[25]
Mo_2_C/NiFe-NC	−51.56 dB/1.4 mm	3.7 GHz/1.4 mm	[41]
Mo_2_C/C composites	−20.38 dB/1.8 mm	5.04 GHz/1.8 mm	[34]
Mo_2_C/C-rGO	−30.00 dB/1.6 mm	5.12 GHz/1.6 mm	[42]
Mo_2_C/La_0_._6_Sr_0_._4_MnO_3_	−39.00 dB/1.2 mm	5.40 GHz/1.2 mm	[43]
Mo_2_C/MoO_2_/C	−62.9 dB/1.9 mm	6.20 GHz/1.9 mm	[44]

## Data Availability

The original contributions presented in the study are included in the article/Supplementary Material, further inquiries can be directed to the corresponding authors.

## Supplementary Materials

The following supporting information can be downloaded at: https://www.mdpi.com/article/10.3390/ma18071573/s1, Figure S1: TG and DTG curves of PCS@MoS_2_.
