# Supplementary material for: Precursor-Derived Mo2C/SiC Composites with a Two-Dimensional Sheet Structure for Electromagnetic Wave Absorption
Source: Materials (Basel). 2025 Mar 31;18(7):1573. doi: 10.3390/ma18071573 (PMC11990315; doi:10.3390/ma18071573)
Supplement: Supplementary file 1 [file materials-18-01573-s001.zip › materials-3515984-supplementary.pdf]

Support Information

Precursor-derived Mo_2C/SiC composites with a two-dimensional sheet structure for electromagnetic wave absorption

Yang Li [a], *Wen Yang* [b,*], *Jipeng Zhang* [b], *Yongzhao Hou* [a, c, d,*], *Guangwu Wen* [a], *Guodong Xin* [c], *Meixian Jiang* [d] *and Yongqiang Ma* [d]

a School of Materials Science and Engineering, Shandong University of Technology, Zibo 255000, China.

b School of Transportation and Verhicle Engineering, Shandong University of Technology, Zibo 255000 China

c Weifang Kaihua Silicon Carbide Micropowder Co.,Ltd, China

d Yantai Glass Coating Micro-Nano Imprinting Technology Innovation Center, Conor Glass Science&Technology Co.,ltd, Yantai 265700 China

*Correspondence: houyz1990@sdut.edu.cn, yangwen004@sdut.edu.cn

During 200-400°C stage, some small molecules in PCS do not participate in cross-linking and curing, and will be removed by gas. From the DTG curve in Figure S1, the quality loss of the attachment is the fastest at 380-390°C DTG curves, which attributed to the low molecular weight oligomers. In the range of 400-600°C, the PCS molecular chain breaks down to form smaller molecular chain fragments, especially in the 520-500°C in DTG curve. At 600-800°C, the branched chain in PCS will break and generate methane (CH_4), which derived from the cleavage of -Si-CH_3 on the branched chain of PCS.

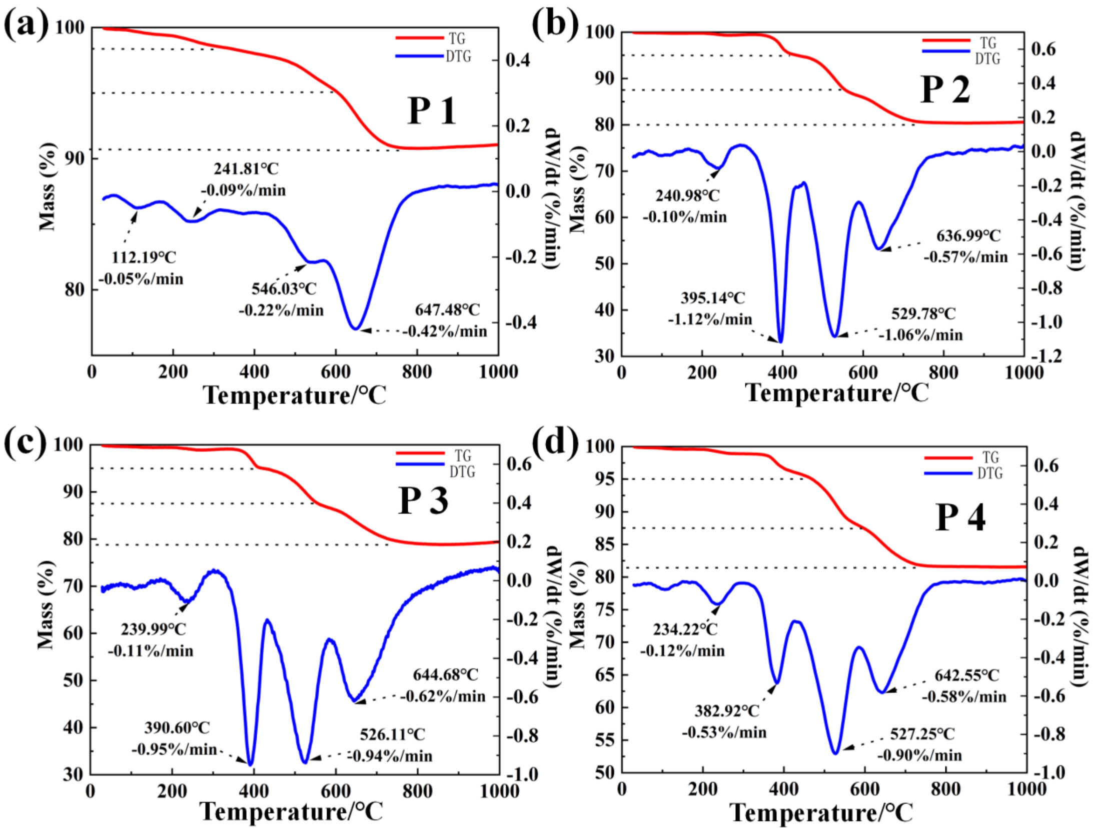

Figure S1 TG and DTG curves of PCS@MoS_2
